# 88 Tubulointerstitial nephropathy and uveitis (TINU syndrome): a paediatric case report

**DOI:** 10.1093/rheumatology/keac496.084

**Published:** 2022-10-07

**Authors:** Hassan Mouffouk, Djohra Hadef, Widad Hezzil, Nacera Bahamma, Asma Guedjiba, Mohamed Fateh Rougui

**Affiliations:** 1 Department of Paediatrics, CHU of Batna; 2 Department of Ophthalmology, CHU of Batna

## Abstract

**Introduction:**

TINU (Tubulo-interstitial Nephritis and Uveitis) or oculo-renal syndrome was first described by Dobrin *et al.* in 1975. >100 cases (mostly paediatric) have been reported since then. It should be considered as diagnosis of elimination, as some iatrogenic, infectious, or systemic diseases may cause similar oculo-renal manifestations. There is no clear therapeutic consensus. However, the rapid initiation of high-dose corticosteroid therapy was associated with favorable outcome in the majority of published cases, as well as in our patient.

**Case report:**

We report the case of an acute tubulointerstitial nephritis associated with uveitis in 11-year-old girl who presented with abdominal pain, vomiting and asthenia of one month. Laboratory tests revealed acute renal failure with proteinuria and aseptic leucocyturia. An inflammatory syndrome was found with erythrocyte sedimentation rate of 92 mm and inflammatory anaemia. Hypoproteinemia and polyclonal hypergamma-globulinaemia was found. No aetiology was found. The renal biopsy revealed a tubulo-nephritis with an interstitial inflammatory infiltrate of lymphoid cells and normal glomeruli. The patient was treated with high-dose of steroids (Methylprednisolone pulse followed by oral Prednisone 1 mg/kg/d with gradual weaning). We noted a progressive improvement of the renal function and the appearance of ocular pain and a decrease in visual acuity. The ophthalmological examination revealed bilateral uveitis. The diagnosis of TINU syndrome was confirmed and the patient received steroids with progressive resolution of the uveitis.

**Conclusion:**

In the presence of any uveitis of unknown cause associated with disturbed renal function the diagnosis of TINU syndrome should be considered. This syndrome requires more precise diagnostic criteria and standardized management.

**Disclosure of Interest:**

None declared

